# Clindamycin-Resistant Clone of *Clostridium difficile* PCR Ribotype 027, Europe

**DOI:** 10.3201/eid1409.071346

**Published:** 2008-09

**Authors:** Denise Drudy, Bram Goorhuis, Dennis Bakker, Lorraine Kyne, Renate van den Berg, Lynda Fenelon, Seamus Fanning, Edward J. Kuijper

**Affiliations:** University College Dublin, Dublin, Ireland (D. Drudy, L. Kyne, L. Fenelon, S. Fanning); Leiden University Medical Center, Leiden, the Netherlands (B. Goorhuis, D. Bakker, R. van den Berg, E.J. Kuijper); European Centre for Disease Prevention and Control, Stockholm, Sweden (E.J. Kuijper)

**Keywords:** Hypervirulent *Clostridium difficile*, Clindamycin Resistance, *ermB*, letter

**To the Editor:** Since 2003, outbreaks of *Clostridium difficile–*associated disease (CDAD) associated with the emergence of a hypervirulent strain have been reported worldwide (*1,2*; www.eurosurveillance.org/em/v12n06/1206-221.asp). This strain has been associated with increased disease severity and attributable mortality. Patients infected with *C. difficile* 027 fail to respond to metronidazole therapy (*1*). Several typing methods have been applied to further characterize *C. difficile* PCR ribotype-027, including pulsed-field gel electrophoresis (PFGE) (North American pulsed field type 1) and restriction enzyme analysis (REA) (BI). PFGE and REA are widely used in the United States; PCR ribotyping is more commonly used throughout Europe. More recently, 2 multiple-locus variable-number tandem-repeat analysis (MLVA) protocols have been applied to type *C. difficile,* and these proved more discriminatory compared to other methods (*3*,*4*). Furthermore, MLVA can subgroup geographically diverse 027 isolates (G. Killgore et al., unpub data) as well as 027 isolates that are common to 1 institution (*5*).

We reported a case of *C. difficile* PCR 027 in Ireland, where the isolate had an identical antibiogram profile compared with those strains reported across Europe (*6*,*7*) (i.e., resistant to fluoroquinolones and erythromycin, susceptible to clindamycin). We have subsequently identified *C. difficile* 027 in 6 more healthcare settings. To date >100 Irish *C. difficile* 027 isolates have been characterized by analysis of their antibiogram profiles, toxinotyping, and 16S–23S rDNA PCR ribotyping. All *C. difficile* 027 isolates were resistant to moxifloxaxin, gatifloxacin, ciprofloxacin (MIC >32 mg/L), and erythromycin (MIC >256 mg/L) but susceptible to metronidazole (MIC 0.25 mg/L) and vancomycin (MIC >0.5 mg/L). Clindamycin susceptibility varied between isolates from unrelated institutions. Isolates from 2 healthcare settings were susceptible to clindamycin (n = 11: MIC_90_ 4 mg/L). However, clindamycin-resistant PCR 027 isolates (n = 96: MIC_90_ >256 mg/L) were identified in the other 5 healthcare institutions. All clindamycin-resistant PCR 027 isolates were positive for the *erm*B gene, encoding the macrolide-lincosamide-streptogramin-B genotype.

A subset of clindamycin-sensitive and -resistant Irish 027 strains isolated throughout 2006 (n = 22) were further characterized by using a recently described MLVA protocol (*3*). Six clindamycin-susceptible isolates were selected from 2 healthcare settings. One hospital conducted active routine laboratory surveillance and molecular genotyping (n = 3). The second hospital submitted only random isolates (n = 3) for typing during a *C. difficile* outbreak. Sixteen clindamycin-resistant PCR 027 isolates were also included in the MLVA. Resistant isolates were selected from 5 healthcare settings. These included isolates from 2 *C. difficile* outbreaks with ongoing laboratory surveillance (n = 5, n = 6, respectively); a third hospital with ongoing laboratory surveillance (n = 3) and 2 hospitals that each submitted fecal samples from patients with severe cases of *C. difficile* disease (n = 1). The Stoke-Mandeville control strain R20291 was included for comparison.

MLVA determined that all strains within the clindamycin-resistant cluster were closely related and were single- or double-locus variants with a maximum 5 summed tandem-repeat difference (STRD). In contrast, the closest relationship between the clindamycin-resistant and the clindamycin-sensitive clusters was a triple-locus variant with an STRD of 17. The nonrelated reference strain of the Stoke-Mandeville outbreak (R20291) differed considerably from all Irish isolates but was more related to the clindamycin-sensitive cluster than to the clindamycin-resistant cluster (Figure). We thus linked a defined genetic marker with the clindamycin-resistant phenotype in *C. difficile* PCR-027. MLVA could clearly differentiate clindamycin-resistant and -susceptible isolates from the same geographic region and subgrouped them into 2 distinct clusters (Figure).

**Figure:**
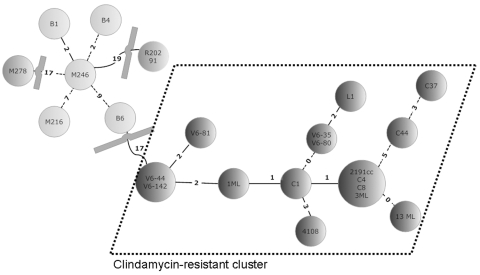
Minimal spanning tree of 23 *Clostridium difficile* isolates. In the circles, the individual isolates are mentioned. The numbers between the circles represent the summed tandem repeat differences (STRDs) between multiple-locus variable-number tandem-repeat analysis types. Straight lines represent single-locus variants, dashed lines double-locus variants. Curved lines represent triple-locus variants. Two related clusters can be discriminated: the light gray cluster (isolates B1, B4, M246, B6, and M216) and the cluster within dotted lines (isolates V6–44, V6–142, V6–81, 1ML, C1, 4108, V6–35, V6–80, L1, 2191cc, C4, C8, 3ML, C44, C37, and 13ML) The isolates in the light gray cluster are sensitive to clindamycin; isolates in the cluster surrounded by dashed lines are resistant. Two isolates (M278 and R20291) did not belong to a cluster but were more related to the sensitive cluster than to the resistant cluster. Genetically related clusters were defined by an STRD <10.

Although high-level resistance to fluoroquinolone antimicrobial agents has been well documented in PCR 027 (*1*,^6^), resistance to clindamycin is rare. Subsequently, clindamycin has been considered as a “protective” antimicrobial agent for the development of CDAD in an epidemiologic survey in the Netherlands (*8*). Currently, resistance to this agent in NAP 1/PCR 027 has been restricted to the United States. McDonald and colleagues reported that 19 (79%) of 24 NAP 1 isolates were classified as less susceptible (MIC 4 mg/L) or resistant (MIC 8 mg/L) to clindamycin when Clinical and Laboratory Standards Institute criteria were used (*2*). Unfortunately, MIC values were not reported, and the corresponding resistance genes were not investigated. In contrast, Canadian studies to date have not reported clindamycin resistance in this strain type. The MIC_90_ of Canadian NAP 1 isolates for clindamycin was 4 mg/L (*9*,*10*). Although outbreaks and sporadic cases of PCR 027 have been identified in several European countries, to date no clindamycin-resistant clone has been reported.

Detection of clindamcyin-resistant *C. difficile* PCR 027 strains is an important and worrying development. Resistance to this antimicrobal agent increases the risk for CDAD in patients, and its use may be an important factor contributing to the persistence and spread of PCR 027. A similar feature has already been observed when fluoroquinolones and cephalosporins are prescribed. Clindamcyin-resistant PCR 027 probably reflects the emergence of a new clone because MLVA clearly differentiates between clindamycin-susceptible and -resistant isolates.

## References

[1] Kuijper EJ, Coignard B, Tull P. the ESCMID Study Group for *Clostridium difficile* (ESGCD)*; EU Member States and the European Centre for Disease Prevention and Control (ECDC). Emergence of *Clostridium difficile-*associated disease in North America and Europe. Clin Microbiol Infect. 2006;12:2–18. 10.1111/j.1469-0691.2006.01580.x 16965399

[2] McDonald LC, Killgore GE, Thompson A, Owens RC Jr, Kazakova SV, Sambol SP, An epidemic, toxin gene-variant strain of *Clostridium difficile.* N Engl J Med. 2005;353:2433–41. 10.1056/NEJMoa051590 16322603

[3] van den Berg RJ, Schaap I, Templeton KE, Klaassen CH, Kuijper EJ. Typing and subtyping of *Clostridium difficile* isolates by using multiple-locus variable-number tandem-repeat analysis. J Clin Microbiol. 2007;45:1024–8. 10.1128/JCM.02023-06 17166961 PMC1829118

[4] Marsh JW, O’Leary MM, Shutt KA, Pasculle AW, Johnson S, Gerding DN, Multilocus variable-number tandem-repeat analysis for investigation of *Clostridium difficile* transmission in hospitals. J Clin Microbiol. 2006;44:2558–66. 10.1128/JCM.02364-05 16825380 PMC1489528

[5] Fawley WN, Freeman J, Smith C, Harmanus C, van den Berg RJ, Kuijper EJ, Use of highly discriminatory fingerprinting to analyze clusters of *Clostridium difficile* infection cases due to epidemic ribotype 027 strains. J Clin Microbiol. 2008;46:954–60. 10.1128/JCM.01764-07 18216211 PMC2268363

[6] Long S, Fenelon L, Fitzgerald S, Nolan N, Burns K, Hannan M, First isolation and report of clusters of *Clostridium difficile* PCR 027 cases in Ireland. Eurosurveillance 2007;12:E070426.3. 10.2807/esw.12.17.03183-en 17868610

[7] Drudy D, Kyne L, O’Mahony R, Fanning S. *GyrA* mutations in fluoroquinolone-resistant *Clostridium difficile* PCR-027. Emerg Infect Dis. 2007;13:504–5. 17552115 10.3201/eid1303.060771 PMC2725882

[8] Goorhuis A, Van der Kooi T, Vaessen N, Dekker FW, Van den Berg R, Harmanus C, Spread and epidemiology of *Clostridium difficile* polymerase chain reaction ribotype 027/toxinotype III in The Netherlands. Clin Infect Dis. 2007;45:695–703. 10.1086/520984 17712752

[9] Bourgault AM, Lamothe F, Loo VG, Poirier L; CDAD-CSI Study Group. In vitro susceptibility of *Clostridium difficile* clinical isolates from a multi-institutional outbreak in Southern Québec, Canada. Antimicrob Agents Chemother. 2006;50:3473–5. 10.1128/AAC.00479-06 17005836 PMC1610058

[10] MacCannell DR, Louie TJ, Gregson DB, Laverdiere M, Labbe AC, Laing F, Molecular analysis of *Clostridium difficile* PCR ribotype 027 isolates from Eastern and Western Canada. J Clin Microbiol. 2006;44:2147–52. 10.1128/JCM.02563-05 16757612 PMC1489423

