# Erratum—Vol. 14, No. 9

**DOI:** 10.3201/eid1410.071196d

**Published:** 2008-11

In Clindamycin-Resistant Clone of *Clostridium difficile* PCR Ribotype 027, Europe (D. Drudy et al.), the Figure contained errors. The correct version appears in the online version of this article (available from www.cdc.gov/EID/content/14/9/1485.htm) and is reprinted below.

We regret any confusion these errors may have caused.

**Figure:**
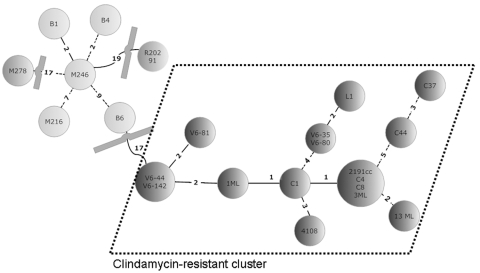
Minimal spanning tree of 23 *Clostridium difficile* isolates. In the circles, the individual isolates are mentioned. The numbers between the circles represent the summed tandem repeat differences (STRDs) between multiple-locus variable-number tandem-repeat analysis types. Straight lines represent single-locus variants, dashed lines double-locus variants. Curved lines represent triple-locus variants. Two related clusters can be discriminated: the light gray cluster (isolates B1, B4, M246, B6, and M216) and the cluster within dotted lines (isolates V6–44, V6–142, V6–81, 1ML, C1, 4108, V6–35, V6–80, L1, 2191cc, C4, C8, 3ML, C44, C37, and 13ML) The isolates in the light gray cluster are sensitive to clindamycin; isolates in the cluster surrounded by dashed lines are resistant. Two isolates (M278 and R20291) did not belong to a cluster but were more related to the sensitive cluster than to the resistant cluster. Genetically related clusters were defined by an STRD <10.

